# Subretinal Implantation of Human Primary RPE Cells Cultured on Nanofibrous Membranes in Minipigs

**DOI:** 10.3390/biomedicines10030669

**Published:** 2022-03-14

**Authors:** Lyubomyr Lytvynchuk, Annabelle Ebbert, Hana Studenovska, Richárd Nagymihály, Natasha Josifovska, David Rais, Štěpán Popelka, Lucie Tichotová, Yaroslav Nemesh, Jana Čížková, Jana Juhásová, Štefan Juhás, Pavla Jendelová, Janka Franeková, Igor Kozak, Slaven Erceg, Zbynek Straňák, Brigitte Müller, Zdenka Ellederová, Jan Motlík, Knut Stieger, Taras Ardan, Goran Petrovski

**Affiliations:** 1 Department of Ophthalmology, Justus Liebig University Giessen, University Hospital Giessen and Marburg GmbH, 35392 Giessen, Germany; brigitte.mueller@augen.med.uni-giessen.de (B.M.); knut.stieger@uniklinikum-giessen.de (K.S.); 2 Karl Landsteiner Institute for Retinal Research and Imaging, 1030 Vienna, Austria; 3 Institute of Macromolecular Chemistry, Academy of Sciences of the Czech Republic, 16206 Prague, Czech Republic; studenovska@imc.cas.cz (H.S.); david.rais@atlas.cz (D.R.); popelkast@centrum.cz (Š.P.); 4 Center for Eye Research, Department of Ophthalmology, Oslo University Hospital and Institute for Clinical Medicine, Faculty of Medicine, University of Oslo, 0450 Oslo, Norway; enmihaly@gmail.com (R.N.); natasha.josifovska@medisin.uio.no (N.J.); goran.petrovski@medisin.uio.no (G.P.); 5 Institute of Animal Physiology and Genetics, Academy of Sciences of the Czech Republic, 27721 Libechov, Czech Republic; tichotova@iapg.cas.cz (L.T.); nemesh@iapg.cas.cz (Y.N.); cizkova@iapg.cas.cz (J.Č.); juhasova@iapg.cas.cz (J.J.); juhas@iapg.cas.cz (Š.J.); ellederova@iapg.cas.cz (Z.E.); motlik@iapg.cas.cz (J.M.); ardan@iapg.cas.cz (T.A.); 6 Department of Cell Biology, Faculty of Science, Charles University, 11000 Prague, Czech Republic; 7 Institute of Experimental Medicine, Academy of Sciences of the Czech Republic, 11720 Prague, Czech Republic; pavla.jendelova@iem.cas.cz (P.J.); serceg@cipf.es (S.E.); 8 Institute for Clinical and Experimental Medicine, 14021 Prague, Czech Republic; janka.franekova@ikem.cz; 9 Moorfields Eye Hospital, Abu Dhabi 62807, United Arab Emirates; igor.kozak@moorfields.ae; 10 Research Center “Principe Felipe”, Stem Cell Therapies in Neurodegenerative Diseases Laboratory, 46012 Valencia, Spain; 11 Department of Ophthalmology, University Hospital Kralovske Vinohrady and Third Faculty of Medicine, Charles University, 10034 Prague, Czech Republic; zbynek.stranak@fnkv.cz

**Keywords:** subretinal implantation, human primary RPE, nanofibrous PDLLA membranes, minipigs

## Abstract

Purpose: The development of primary human retinal pigmented epithelium (hRPE) for clinical transplantation purposes on biodegradable scaffolds is indispensable. We hereby report the results of the subretinal implantation of hRPE cells on nanofibrous membranes in minipigs. Methods: The hRPEs were collected from human cadaver donor eyes and cultivated on ultrathin nanofibrous carriers prepared via the electrospinning of poly(L-lactide-co-DL-lactide) (PDLLA). “Libechov” minipigs (12–36 months old) were used in the study, supported by preoperative tacrolimus immunosuppressive therapy. The subretinal implantation of the hRPE-nanofibrous carrier was conducted using general anesthesia via a custom-made injector during standard three-port 23-gauge vitrectomy, followed by silicone oil endotamponade. The observational period lasted 1, 2, 6 and 8 weeks, and included in vivo optical coherence tomography (OCT) of the retina, as well as post mortem immunohistochemistry using the following antibodies: HNAA and STEM121 (human cell markers); Bestrophin and CRALBP (hRPE cell markers); peanut agglutining (PNA) (cone photoreceptor marker); PKCα (rod bipolar marker); Vimentin, GFAP (macroglial markers); and Iba1 (microglial marker). Results: The hRPEs assumed cobblestone morphology, persistent pigmentation and measurable trans-epithelial electrical resistance on the nanofibrous PDLLA carrier. The surgical delivery of the implants in the subretinal space of the immunosuppressed minipigs was successfully achieved and monitored by fundus imaging and OCT. The implanted hRPEs were positive for HNAA and STEM121 and were located between the minipig’s neuroretina and RPE layers at week 2 post-implantation, which was gradually attenuated until week 8. The neuroretina over the implants showed rosette or hypertrophic reaction at week 6. The implanted cells expressed the typical RPE marker bestrophin throughout the whole observation period, and a gradual diminishing of the CRALBP expression in the area of implantation at week 8 post-implantation was observed. The transplanted hRPEs appeared not to form a confluent layer and were less capable of keeping the inner and outer retinal segments intact. The cone photoreceptors adjacent to the implant scaffold were unchanged initially, but underwent a gradual change in structure after hRPE implantation; the retina above and below the implant appeared relatively healthy. The glial reaction of the transplanted and host retina showed Vimentin and GFAP positivity from week 1 onward. Microglial activation appeared in the retinal area of the transplant early after the surgery, which seemed to move into the transplant area over time. Conclusions: The differentiated hRPEs can serve as an alternative cell source for RPE replacement in animal studies. These cells can be cultivated on nanofibrous PDLLA and implanted subretinally into minipigs using standard 23-gauge vitrectomy and implantation injector. The hRPE-laden scaffolds demonstrated relatively good incorporation into the host retina over an eight-week observation period, with some indication of a gliotic scar formation, and a likely neuroinflammatory response in the transplanted area despite the use of immunosuppression.

## 1. Introduction

Age-related macular degeneration (AMD) is one of the most prevalent forms of irreversible visual impairment with multifactorial etiology. Its pathogenesis is characterized by the early appearance of drusen, followed by functional anomalies in the retinal pigmented epithelium (RPE) and geographic atrophy, followed by photoreceptor degeneration affecting the macular region [1].

There are multiple reasons for the photoreceptor dysfunction and degeneration, including the ionization of photosensitive retinoids upon light exposure, the generation of reactive oxygen species and inflammatory cascade, leading to photoreceptor cells death. Among the major factors leading to photoreceptor cell death are those associated with an increased permeability of RPE, aberrant choroidal neovascularization [2], altered RPE phagocytosis [3], as well as sub-retinal recruitment of resident and monocytic microglial cells [4]. Functional as well as structural changes in the RPEs are thought to drive the onset of the disease. RPEs are specialized pigmented cells arranged in a compact monolayer between the neural retina and the choriocapillaris. The loss or degeneration of the RPEs results in vision loss due to their important function in supplying nutrients to the photoreceptors (rods and cones), their phagocytosis and maintenance of the blood–retina barrier among other factors [5]. The cause of degeneration of the RPEs has not been fully deciphered to date. There are no therapeutic interventions that can reverse AMD [6,7]. However, treatment modalities such as the injection of anti-vascular endothelial growth factors (VEGFs), laser-based coagulation or photodynamic therapy of the neovascularisations have been used to reduce the severity of the wet form of AMD [8]. The cell therapy-based regenerative approach offers a solution in treating and reversing AMD [9]. Stem cell-derived sources can be used for the derivation of allogenic or autologous induced RPEs (iRPE) that can be injected into the subretinal space to regenerate the lost RPE monolayer and thus reduce the symptoms of AMD, including vision loss [10,11,12,13]. However, recent studies have suggested that the injection of RPEs in the subretinal space leads to the incomplete epithelization of the degenerated RPE monolayer, as well as suboptimal cell engraftment and a diminished reversibility of vision loss. This could be attributed to various reasons such as the improper attachment of the RPEs on the Bruch’s membrane, loss of cell-to-cell contact between the RPEs, change in functional behaviour of transplanted RPEs in the diseased environment, and de-differentiation of transplanted RPEs leading to the loss of functionally mature cells [7]. To overcome this limitation, RPEs seeded on a bioscaffold were transplanted and demonstrated to improve the integration, regeneration and functional behaviour in vivo upon transplantation, as compared to the cell-injection method of delivery. Such knowledge further accentuates the need for newer scaffold-based delivery approaches, wherein RPEs pre-seeded patches could be directly transplanted into the subretinal space [14]. This would resolve some of the limitations observed in cell delivery modalities including the de-differentiation of RPEs, and help with the proper engraftment of the transplanted RPEs, with eventual complete degradation of the scaffold and monolayer cellularization of the RPE layer.

The aforementioned strategies are clinically viable, if a reproducible, efficient and faster way of deriving the already dedicated RPEs from human cadaver donors exists. So far, most studies have focused on the in vitro development of iPSCs involved in the spontaneous derivation of RPE-like cells during the culturing of ectoderm-committed embryonic stem cells (ESCs). This process usually takes 4–6 months to achieve, providing a sufficient number of cells for in vivo studies [7,11,13]. Furthermore, the process is spontaneous and factors such as efficiency, gene and the functional level of RPE remain elusive and may vary from batch to batch.

In order to increase and maintain the differentiation state, strategies and improvements have been made to achieve a better yield of RPEs derived from ESCs and iPSCs using either biphasic or triphasic methods [7,14,15,16], however, still in a rather complex and lengthy manner. Alternatively, differentiated human primary RPEs (hRPEs) from cadaver donors can be used as a potential source to derive cellular therapy modalities and applications in disease modelling, as well as for in vitro development for drug testing. The development of hRPEs for transplantation purposes on biodegradable scaffolds for human transplantation is therefore indispensable. Herein, we present the initial methodology and the results of such an approach.

## 2. Materials and Methods

### 2.1. Ethics

This study was performed in accordance with the Guidelines of the Declaration of Helsinki and complied with the The Association for Research in Vision and Ophthalmology (ARVO) statement for the use of animals in ophthalmic and visual research. The study protocol regarding the isolation and cultivation of hRPE cells was approved by the Regional Committees for Medical and Health Research Ethics for the Center for Eye Research, Department of Ophthalmology, Oslo University Hospital and University of Oslo (Oslo, Norway) with Ref. nr.: 2017/418. The study protocol, which included an experimental animal study, was approved by the Ethical Committee of Institute of Animal Physiology and Genetics, Academy of Sciences of the Czech Republic (Libechov, Czech Republic) about defending animals from violence (No. 60/2016). The study was performed as part of the international project of The Czech Science Foundation (Project Number 18-04393S) and Technology Agency of the Czech Republic (KAPPA project TO01000107).

### 2.2. Isolation and Cultivation of hRPEs

The hRPEs were collected from human cadaver donor eyes following the Guidelines of the Declaration of Helsinki and approved by the Regional Committees for Medical and Health Research Ethics, Norway (REK: 2017/418). The hRPEs were isolated enzymatically, using trypsin, over a period 30 min (Gibco^®^, Thermo Fisher Scientific, Waltham, MA, USA) or by gently scraping the RPE layer without damaging the Bruch’s membrane using half spherically bent-end Pasteur glass pipettes according to a previously described procedure [3]. The cells were passaged by incubation in TrypLE (Gibco^®^) for 5 min. The hRPE cells (passage 0) were cultured in 10% fetal bovine serum (FBS) for up to 14 days to reach confluency, with the medium changed to 1% FBS. The seeding of the hRPEs was performed in trans-well plates containing a custom-made ultrathin nanofibrous carrier at a density of 2000 cells/mm^2^ and incubated for an additional 30 days before transplantation.

### 2.3. Ultrathin Nanofibrous Carrier for RPE Transplantation

**Figure 1:**
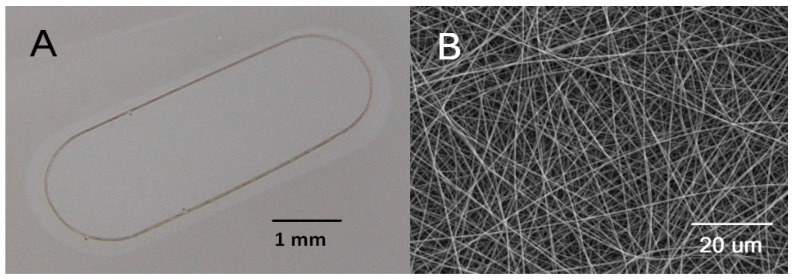
Nanofibrous carrier with embedded supporting PET frame. The carrier was cut from a cell culture insert with a modified punch. Three visible marks on the frame allow to control a side orientation of the carrier (**A**). Electron microscopy of the nanofibrous membrane (**B**).

Nanofibrous carriers were prepared by the electrospinning of poly(L-lactide-co-DL-lactide) (LLA/DLLA 90/10, Mw 868 270 g/mol, PDI 2.3). The thin nanofibrous membrane was reinforced with a peripheral frame, which was embedded in the membrane during electrospinning following a previously published procedure [17] (Figure 1).

The diameter of the bead-free fibres was 380 nm (standard deviation, SD 10 nm) and the average pore size of the membrane was 0.4 µm (SD 0.2 µm). The membrane was 3.7 µm in thickness and its porosity was estimated to be 72% [18].

The supporting oval frames were cut from a bi-axially oriented poly (ethylene terephthalate) (PET) foil, 36 µm in thickness, using a laboratory-assembled femtosecond laser microfabrication station. Each frame was equipped with a mark for side orientation.

The sheets of the membrane were fixed to the body of commercial cell-culture insert Falcon (Corning Inc., Kenneburg, ME, USA) to facilitate the seeding and growth of the cells. Samples were air-plasma treated for 30 s at the power of 7 W prior to RPE cultivation. The carriers 5.2 mm × 2.1 mm, with an adherent sheet of RPE cells, were cut using a shaped biopsy punch prior to loading into the implantation injector.

### 2.4. Trans-Epithelial Electrical Resistance (TEER)

Nanofibrous membranes were coated using 5 μg/mL laminin overnight to assist cell attachment. The following day, hRPE cells were seeded at passage 1 onto the membranes at a density of 2000 cells/mm^2^ and cultured on the membranes for up to 6 weeks until TEER measurements took place. A Millicell^®^ ERS-2 Voltohmmeter (Merck, Darmstadt, Germany) was used to determine the membrane resistance values. Cultured cells were provided with a fresh, serum-free medium and an internal control electrode was used to calibrate the device prior to measurement. The electrodes, which were small enough to fit between the trans-well inserts, were dipped into the wells following the manufacturer’s guidelines and the data were recorded.

### 2.5. Implantation Injector

The custom-made injector consisted of a rectangular plastic capillary (outer dimensions 2.8 × 0.8 mm) and a stainless-steel blade plunger. The length of the plastic capillary was 6 cm. The carrier was loaded into the injector through a loading window located 6 mm from the distal end of the capillary in sterile conditions in the experimental department of the laboratory facility. The injector with preloaded carriers was prepared separately for each case. The injectors were transferred to the operating theatre in a sterile container shortly before the implantation stage of the surgery in order to avoid the drying up of the carrier, which could cause the adhesion of the carrier to the inner surface of the capillary. Before the implantation stage of the surgery, the injector was taken out of the container and inserted through the large sclerotomy into the vitreous cavity. After the surgeon performed the injection of the carrier into the subretinal space by pushing the plunger, the injector was withdrawn from the eye.

### 2.6. Animals Used for the Experimental Study

“Libechov” minipigs that were 12–36 months old and of both sexes were used in the study [19]. All minipigs used in this study were wild-type. General anaesthesia of the minipigs was induced by intramuscular injection of a TKX mixture composed of Tiletamine (2 mg/kg, Zoletil 100, Virbac, Carlos, France), Zolazepam (2 mg/kg, Zoletil 100, Virbac), Ketamine (2 mg/kg, Narketan 10, Vetoquinol, Lure, France), and Xylazine (0.4 mg/kg, Xylapan, Vetoquinol). After the induction of sedation, an ear vein cannula was introduced and the animal was intubated for inhalation maintenance of anaesthesia (Isoflurane 1.5%). During eye surgery, intramuscular injection was administered to the animals by Eficur (1 mL/16 kg BW, Hipra, Girona, Spain) and Depo-Medrol 120 mg (Pfizer, New York, NY, USA). Eficur was repeatedly injected 24 and 48 h after surgery, followed by a 72 h injection of the animals by Draxxin (1 mL/40 kg BW, Zoetis, Parsippany, NJ, USA) as a secondary bacterial infection prevention. Postoperatively, Ophthalmo-Framycoin ointment (Zentiva, Prague, Czech Republic) was applied to the conjunctival sac of the animals five times per day during 1 week. Animals were sacrificed by exsanguination in deep general anaesthesia 1, 2, 6, and 8 weeks after RPE cell-graft implantation.

### 2.7. Immunosuppression of Minipigs

**Figure 2:**
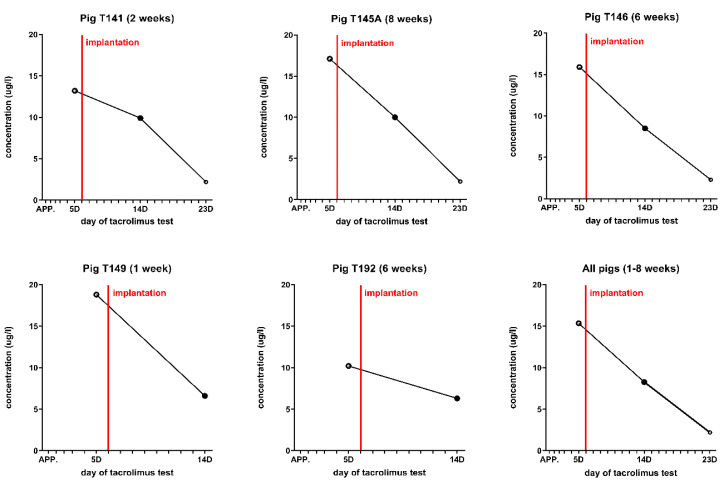
Tacrolimus blood levels measured throughout the experimental studies in minipigs. The concentration of tacrolimus in the blood was examined on Days 5, 14, and 23 after initial application.

The survival of the donor cells of the recipient animals was supported by a single subcutaneous injection of tacrolimus-eluting polymer microspheres. We used tacrolimus in a dose of 0.25 mg/kg BW. Tacrolimus-eluting polymer microspheres were prepared from tacrolimus powder (Prograf^®^, Astellas Pharma, Deerfield, IL, USA) and poly(D,L-lactide-co-glycolide) (Resomer RG 503H, Evonik, Germany) by adopting a procedure previously described by Wang et al. (Citation). The concentration of tacrolimus in polymer microspheres was 51.3 mg/g, as determined by HPLC. The proper application of tacrolimus-containing microspheres was performed six days before eye surgery and only once at a time. The tacrolimus levels in the blood of all minipigs are shown in Figure 2.

### 2.8. Implantation of hRPEs on a Nanofibrous Carrier into Minipigs

Five minipigs treated with tacrolimus-eluting polymer microspheres were used throughout the experiment and followed up to 8 weeks (Table 1).

A nanofibrous membrane without RPE cells was implanted into the subretinal space of a minipig using the same surgical procedure and an implantation injector. After 4 weeks, the minipig underwent euthanasia. For histological evaluation, the enucleated eye was fixed in 4% paraformaldehyde for 24 h and embedded in paraffin.

### 2.9. Surgical Technique

All surgeries were performed in the operating room of the Institute of Animal Physiology and Genetics, Academy of Sciences of the Czech Republic (Libechov, Czech Republic) by three experienced vitreoretinal surgeons (L.L., G.P., Z.S.) who previously accomplished a training course and obtained a certificate, which permits them to perform eye microsurgeries on pigs for experimental objectives. Every surgical procedure was performed under general anaesthesia, with the implementation of standard aseptic and antiseptic measurements. A microsurgical approach was facilitated using an ophthalmic surgical microscope Hi-R NEO 900A (Haag-Streit Surgical, Wedel, Germany), vitrectomy machine R-Evolution CR (Optikon, Rome, Italy) and a non-contact indirect viewing system MERLIN BIOM (Volk, Mentor, OH, USA). After the skin and the conjunctival sac were disinfected using 10% and 5% solution of povidone–iodine (Betadine), respectively, the speculum was inserted. The conjunctiva was opened via the nasal, 2.5 mm from the limbus and was 5 mm long. Episcleral vessels were cauterized. A standard three-port vitrectomy was conducted with 23-gauge (G) trocars, which were inserted 2.5–3 mm from the limbus in a standard position (Supplementary Figure S1). Core pars plana vitrectomy (PPV) was performed via the removal of the vitreous centrally and anterior to the equator at the site of the future insertion of the injector. Posterior vitreous detachment was assisted by the intravitreal injection of triamcinolone acetonide (TA) crystals. The creation of the subretinal bleb was facilitated via a subretinal injection of a balanced salt solution (BSS^®^, Alcon Laboratories, Inc., Fort Worth, TX, USA) with subretinal PolyTip cannula 25/38 gauge (MedOne Surgical, Inc., Sarasota, FL, USA), which was connected through a Luer lock adapter to the syringe filled with BSS^®^. Subretinal fluid application was performed from the nasal side toward the centre of the retina, so as to avoid the formation of a bleb in the periphery. Caution was taken to avoid the injection of BSS under the RPEs with a detachment of the choroid. During the subretinal application of BSS, the intraocular pressure settings on the vitrectomy machine decreased from 25 to 15 mmHg. After the episcleral vessels were cauterized with exodiathermy, a 3 mm long limbus parallel sclerotomy between the two nasal trocars was performed 2.5 mm from the limbus with a beveled angled Phaco Slit Knife 2.2 mm (D.O.R.C., Zuidland, The Netherlands). After the removal of the prolapsed vitreous, an Injector was introduced into the vitreous cavity and the carrier was injected into the subretinal space through the retinotomy. Following withdrawal of the injector, the 3 mm sclerotomy was sutured with Vicryl 8.0 (Johnson & Johnson, New Brunswick, NJ, USA). A fluid-air exchange was performed under visual control with injection of endotamponade with silicone oil 1000 cSt (D.O.R.C., Zuidland, The Netherlands). The position of the implanted carrier was documented using fundus photography. Trocars were removed, and the sclerotomies and the conjunctival wound were sutured with Vicryl 8.0. The conjunctival sac was washed with Betadine, and the subconjunctival injection of gentamicin 20 mg and dexamethasone 2 mg was performed at the end of the surgery. All surgeries were documented by drawing the fundus scheme and recording in video format (video and snapshot modes).

### 2.10. In Vivo Optical Coherence Tomography

The retina of all animals was examined by spectral-domain optical coherence tomography (SD-OCT) system iVue (Optovue, Fremont, CA, USA).

### 2.11. Euthanasia and Tissue Processing

Euthanasia was performed under general anesthesia with the intramuscular injection of the T61 mixture (Embutramidum 200 mg/kg, Mebezonii iodidum 50 mg/kg, Tetracaine hydrochloride 5 mg/kg, Intervet International B.V, AN Boxmeer, The Netherlands). Whole eyes were removed and fixed in 4% paraformaldehyde (PFA) for 24 h before being stored in 1 × PBS at 4 °C before further processing. The anterior cup of the eye was removed by circumferential incision at the limbus. The eyecup was oriented according to the pig’s visible characteristic major blood vessels, the implanted scaffold identified in the nasal central retina and isolated with the sclera attached. Control eyes were dissected the same way, and the nasal central retina was collected. All tissues were cryoprotected in graded sucrose solutions as described in detail [20]. Vertical sections (14 µm) were cut with a cryomicrotome (SLEE Medical GmbH, Mainz, Germany) and collected on Superfrost slides.

### 2.12. Immunohistochemical Analysis

For frozen sectioning, porcine eyecups were treated as previously described [20]. Immunostaining was performed by employing the two-step indirect method. Sections were incubated at room temperature overnight in primary antibodies (Table 2). Immunofluorescence was performed using Alexa Fluor 488-conjugated secondary antibodies (#21202, Thermo Fisher Scientific, Bremen, Germany) or Alexa 594 (#21207, Thermo Fisher Scientific, Germany) and Alexa 647 (# A-21447, Thermo Fisher Scientific, Germany).

### 2.13. Laser Scanning Confocal Microscopy

Confocal images were captured using an Olympus FV10i confocal microscope, equipped with Argon and HeNe lasers. A high-resolution scanning of image stacks was performed with an UPlanSApo × 60/1.35 (Olympus) oil-immersion objective at 1024 × 1024 pixels and a *z*-axis increment of 0.3 µm. For an analysis of the immunolabelled cells and their processes, a stack of 2–12 sections was used (0.7-µm *z*-axis step size). Cell processes were reconstructed by collapsing the stacks into a single plane. Brightness and contrast of the final images were adjusted using Adobe Photoshop CS5 (San Jose, CA, USA).

## 3. Results

### 3.1. Production of a Monolayer of hRPEs on a Nanofibrous Carrier

**Figure 3:**
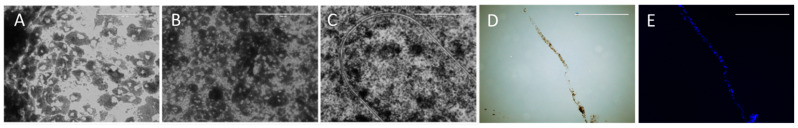
Morphology of the cultured hRPEs on the nanofibrous carrier. (**A**) Typical morphology of the cells between Days 15–44 (20× magnification, scale bar = 200 µm), (**B**) at Day 74 (20× magnification, scale bar = 200 µm), and (**C**) at Day 74 (4× magnification, scale bar = 1 mm). (**D**) Typical morphology of a cross-section of cultured hRPEs (10× magnification, scale bar = 400 µm), also found to be stained by DAPI (**E**).

The hRPEs assumed cobblestone morphology, with full pigmentation maintained throughout the cultivation period on a nanofibrous carrier (Figure 3).

### 3.2. Trans-Epithelial Electrical Resistance (TEER)

The resistance values for two donors of hRPE were determined on two different occasions with a repetition of 3 (*n* = 6). The mean and standard deviation of all measurements for TEER was 128.82 ± 16.45 Ω.cm^2^. Without cells, the electrode recorded an average resistance of 89.85 ± 11.65 Ω at the two sides of the nanofibrous membrane. Corrected for the surface area, the hRPE seeded on the nanofibrous membranes had a resistance of 38.61–75.11 Ω.cm^2^.

### 3.3. Implantation of the hRPEs

**Figure 4:**
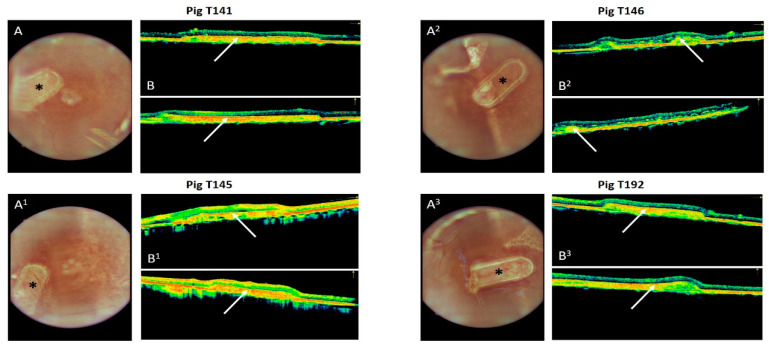
Fundus (**A**) and Optical Coherence Tomography (**B**) imaging of the implanted hRPEs on a nanofibrous carrier into minipigs. (**A**,**A^1^**,**A^2^**,**A^3^**) The star indicates the position of the implant in the superior nasal area of the retina. (**B**,**B^1^**,**B^2^**,**B^3^**) White arrow highlights the location of the implant underneath the retina in two cross-sectional images of the retina. The strong red line indicates the reflection from the RPE, which seems to be thicker in the area of the transplant.

The typical appearance of the fundus of the transplanted hRPEs on a nanofibrous carrier is shown in Figure 4, together with an OCT image of the retina. The images demonstrate the subretinal location of the implants. The adherence of the implants to the surrounding structures as well as their similar hyperreflectivity appearance to the same level layers in the retina is visible on OCT.

### 3.4. Immunohistochemical Analysis of the Implanted hRPEs

**Figure 5:**
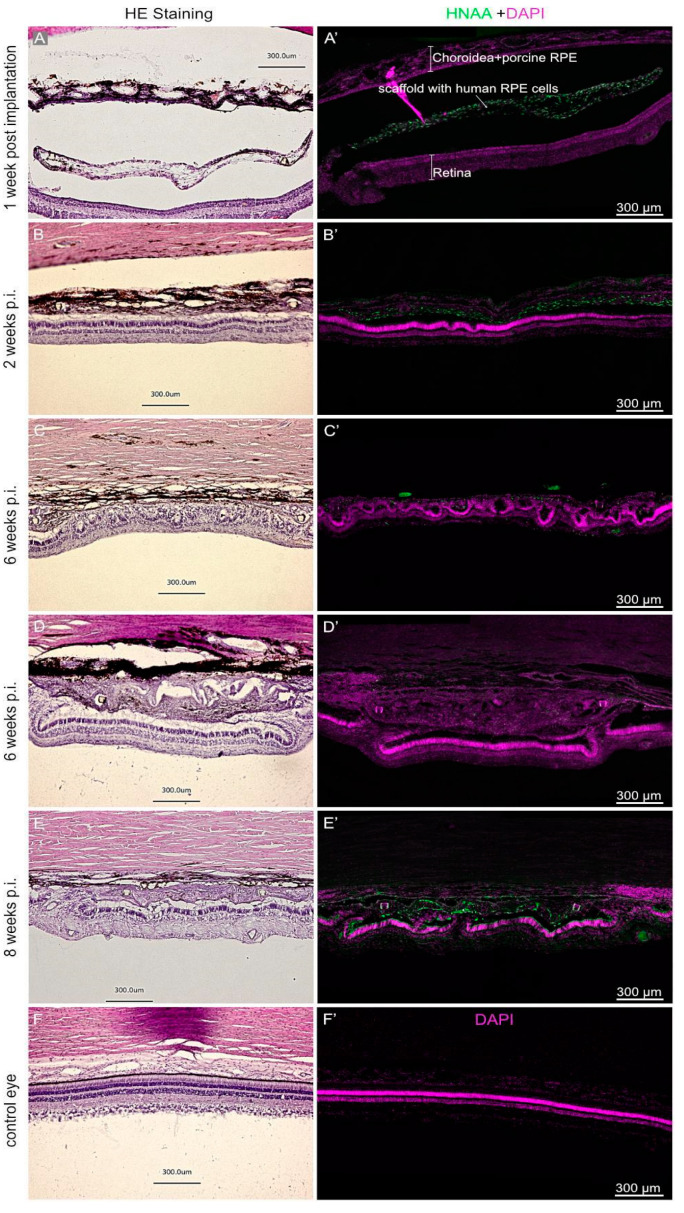
Hematoxylin & eosin staining of the retinal area containing the transplanted cells. Expression of Human Nuclear Antigen (HNAA, green) was observed in hRPE cells at week 1 and 2 post implantation and only occasionally at 6 weeks. Observations are presented at 1 (**A**,**A’**), 2 (**B**,**B’**), 6 (**C**,**C’**,**D**,**D’**), and 8 (**E**,**E’**) weeks as well as a control eye (**F**,**F’**). Nuclear staining was performed by DAPI (purple).

The implanted hRPEs were positive for the human nuclear antigen (HNAA) and were located between the neuroretina and the RPE minipig layer at week 1 after the implantation, this positivity was gradually lost at week 6. The pigmented appearance of the implanted hRPEs could be observed throughout the whole observation period on the H and E stained images as well as in the immunofluorescence images (Figure 5). The melanin granules present in the hRPE cells are highly reflective. However, in one minipig eye, which was enucleated 1 week after the surgery, the nanofibrous membrane with the hRPE cells did not integrate into the subretinal space (Figure 5A,A’). Due to the resulting retinal detachment, we noticed retinal degeneration to a great extent in the photoreceptors. This was apparent in the reduced number of nuclei in the outer nuclear layer. Nevertheless, hRPE cells show strong immunoreactivity for HNAA marker (Figure 5A’). In addition, we observed a rosette formation of the neuroretina under the implants or hypertrophic reaction appearance of tissue in the implanted area at week 6 and partly at week 8 (Figure 5D–F’).

**Figure 6:**
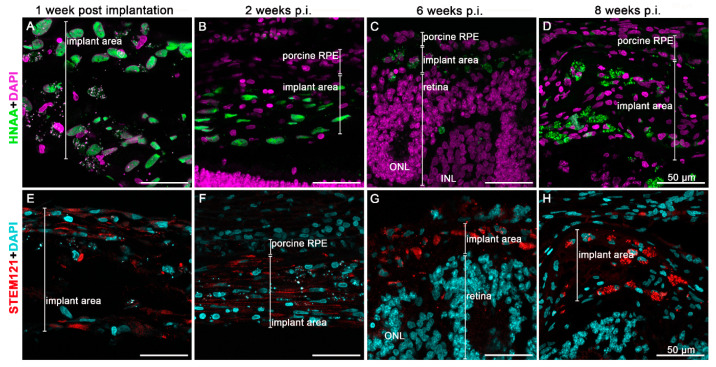
Expression of the human cell markers HNAA (green) and STEM121 (red) on hRPEs implanted on nanofibrous carriers and followed up to 8 weeks. Tissue was stained with HNAA (**A**–**D**) and STEM121 (**E**–**H**). Nuclear staining was performed by DAPI (purple and blue). ONL = outer nuclear layer.

The morphology of the implanted cells appearing not in a monolayer after implantation and followed with the HNAA as well as the STEM121 human cell marker, is shown in Figure 6. Up to week 2, the HNAA displayed uniform nuclear staining (Figure 6A,B). Likewise, the human cell marker STEM121 was clearly detectable in the cytoplasm of hRPE cells up to week 2 (Figure 6E,F). Only very few hRPE cells at week 6 post-transplantation present STEM121 immunoreaction (Figure 6G). Neither of the two human markers detected any hRPE cells at week 8 post-transplantation. Nevertheless, they were still detectable via their strongly fluorescent melanin granules (Figure 6D,H).

**Figure 7:**
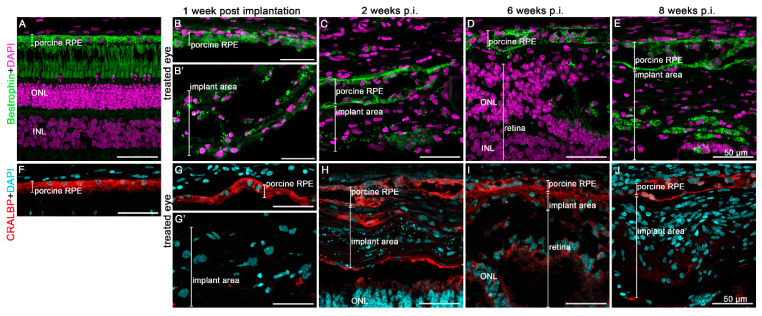
Expression of the RPE cell markers bestropin (**B**–**E**) and cellular retinaldehyde binding protein (CRALBP, red) (**G**–**J**) in the treated minipigs continued for up to 8 weeks. (**A**,**F**) are images of the control eyes to demonstrate specificity of the staining. Immunofluorescence analysis of the given marker and its appropriate color is shown. Nuclear staining was performed by DAPI (purple in (**A**–**E**) and blue in (**F**–**J**)). ONL = outer nuclear layer, INL = inner nuclear layer.

Only the endogenous RPE cells clearly expressed the typical RPE marker bestrophin and CRALBP over time, while the implanted hRPEs did not (Figure 7). However, at 2 weeks post-implantation, individual hRPE cells in the implant area show CRALBP expression (Figure 7H). Bestrophin staining was hardly visible in implanted hRPEs over time (Figure 7B–E). Meanwhile, the transplanted hRPEs appeared to differ significantly from the minipig RPEs—they did not form a confluent layer, and were less capable of keeping the inner and outer retinal segments intact.

**Figure 8:**
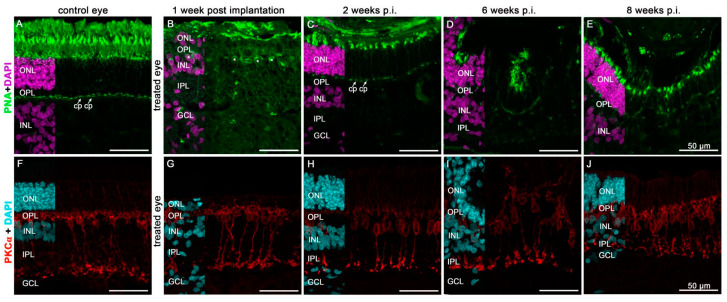
Expression of the cone photoreceptor (peanut agglutinin, PNA) (**B**–**E**) and the rod bipolar (PKCα) (**G**–**J**) markers in the neuroretina underlying the hRPE cell implant followed up to 8 weeks. (**A**,**F**) are images of control eyes to demonstrate specificity of the staining. Immunofluorescence analysis of the given marker and its appropriate color is shown. Nuclear staining was performed by DAPI (purple in (**A**–**E**) and blue in (**F**–**J**)). Asterisks in B mark cone cell bodies that were slightly dislocated in the INL due to retinal detachment. Cp = cone pedicles, ONL = outer nuclear layer, INL = inner nuclear layer, OPL = outer plexiform layer, IPL = inner plexiform layer, GCL = ganglion cell layer.

The cone photoreceptors adjacent to the scaffold seemed to be unchanged (Figure 8B,C,E). However, with as time passed following transplantation, changes in the retinal structure occurred, such as the rosette formation visible six weeks post-implantation (Figure 8D). Nevertheless, even eight weeks post-implantation, all PNA immunopositive compartments of cone photoreceptors could be detected (Figure 8E). The nutrient supply remained stable despite the implanted scaffold—the under lying retina appears relatively healthy. However, the interaction with the implanted cells with regard to the outer-segment formation could not be observed. Inner retinal structures seemed to be better preserved, as seen with the staining of rod bipolar cells (Figure 8F–J).

The cell death of transplanted hRPE cells and underlying neuroretina was verified by TUNEL staining. During the overall time-period of the study, only very few lightly TUNEL-positive nuclei in the implant area were detectable (Supplementary Figure S2). In most cases, they matched the lightly DAPI stained heterochromatin, an indication of DNA degradation. Only occasionally, brightly TUNEL positive nuclei were visible, as was the case in the hypertrophic implant area 6 weeks post-transplantation (Supplementary Figure S2M–O). The retina underneath the implant showed no TUNEL-positive nuclei during the whole period. However, the only exception was the minipig sacrificed 1 week post-implantation with the retinal detachment due to the unsuccessful integration of the implant.

**Figure 9:**
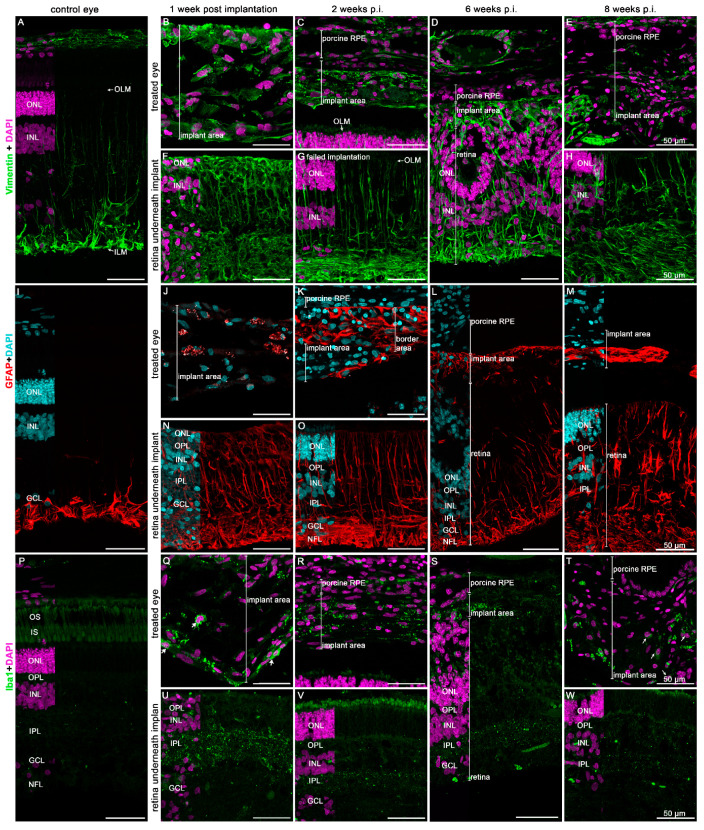
Expression of macroglial (Vimentin, **B**–**H**, GFAP, **J**–**M**) and microglial (Iba1, **Q**–**W**) markers in the neuroretina underlying the hRPE cells implant followed up to 8 weeks. (**A**,**I**,**P**) are images of control eyes to demonstrate specificity of the staining. Immunofluorescence analysis of the given marker and its appropriate color is shown. Nuclear staining was performed by DAPI (purple in (**A**–**H**,**P**–**W**) and blue in (**I**–**M**)). OLM = outer limiting membrane, ONL = outer nuclear layer, INL = inner nuclear layer, OPL = outer plexiform layer, IPL = inner plexiform layer, GCL = ganglion cell layer.

With regard to the glial reaction of the retina to the transplant, GFAP and vimentin markers were evaluated (Figure 9). While the vimentin-positive signal could be observed in the untouched porcine retina to a certain extent, in both the ganglion cell layer and at the level of the inner limiting membrane (Figure 9A), in the transplanted retina, from 1 week onwards throughout the observational period of 8 weeks, vimentin staining could be observed in the entire retina including the transplant itself (Figure 9B–H). Similar observations were found with GFAP (Figure 9I–M), indicating the formation of some level of a gliotic scar within the area of transplantation. Microglial activation was observed in the retinal area of the transplant soon after the surgery (Figure 9Q,U), which moved into the transplant over time (Figure 9T). This indicates a likely neuroinflammatory response in the transplanted area.

## 4. Discussion

### 4.1. RPE Preparation for Transplantation and Post-Implantation Characterization

Several advancements have been made in the field of RPE derivation using iPSCs and ESCs [21,22]; however, no advancement to date has been made on using fully differentiated hRPEs in transplantation studies. ESCs continue to pose ethical issues with regard to their use, whereas iPSCs are patient-specific and can maintain their epigenetic memory [23]. Therefore, in recent years, beside the interest in iPSC- and ESC-based RPE derivation, hRPEs have been frequently applied in the explored methods for clinical trials. The iPSC-derived RPEs have already been transplanted in AMD patients as a cell therapy module or in scaffold-based RPE patches. Results have been positive from these clinical trials [13]. However, such studies have been carried out on a small scale, and can be used as proof-of-concept studies for RPE-based treatment of AMD in humans. Furthermore, iPSC-based RPE derivation in itself has many challenges, including, among others, the long-duration required to differentiate iPSCs into RPEs, which varies between 2 to 6 months [7].

Additionally, iPSC- and ESC- derived RPEs have been used in clinical trials for AMD patients [24]. Recent studies on clinical trials of stem cell-derived RPEs include ESC-derived RPEs used for the treatment of geographic atrophy in AMD [25], ESC-derived RPE patch in AMD patients [26] and iPSC-derived RPE sheets in a patient with AMD. These studies have focused more on the differentiation aspect in order to reduce the differentiation time, which is not an issue when using hRPEs. More recently, a cocktail of small molecule inhibitors and growth factors have been used to derive RPEs and further fabricate a patch for transplantation in a rat and pig model to study the efficiency of RPE patch attachment and the function of the transplanted construct. The commitment and differentiation of these cells is obviously important [27,28].

Our hRPEs possessed full pigmentation during ex vivo cultivation on the nanofibrous carrier, and formed folds after subretinal transplantation. Other studies have shown similar native infoldings [29] when human iPSC-derived RPEs were cultured on nanofibrous membranes, with similar morphological, electrical, and RPE markers’ expression characteristics. In addition, the hRPEs formed rosettes, which have been shown before in a retinal transplantation study [30]. The low thickness and high porosity of our nanofibrous membranes should also positively affect the long-term survival of the hRPEs [31]. Our cells, unlike other previously reported cells [32], as well as stem cells-derived RPEs [14], produced no extracellular deposits on their basal side. Our nanofibrous membrane with a degradation lifetime of at least 5 months or more [33,34,35], and with no adverse effects present during the polymer degradation [36], appeared to be a plausible and sufficient carrier for a typical 2–6 month-long cultivation of RPEs ex vivo [37,38], as well as mechanical manipulation during surgery. The degradable nature of the nanofibrous membrane seemed to also be advantageous in supporting the de novo synthesis of a Bruch’s membrane equivalent by the donor RPE cells, and their integration into the host’s Bruch’s membrane [14]. Our implanted patch of cells contained a peripheral frame [17] around the nanofibrous membrane, which seemed to provide sufficient mechanical strength during the surgical procedure.

The measurement of TEER is used to determine the overall health and confluence of a cell monolayer, and as such, an increased value thus represents a strong barrier against an electric current [39,40]. The relatively low net resistance detected in our cultures (38.61–75.11 Ω.cm^2^) may indicate an incomplete formation of tight junctions and cell polarization. The resistance of the native RPE layer is believed to be around 150 Ω.cm^2^; however, the values range between 25–500 Ω.cm^2^ with cultured RPE cells. Improvements in the hRPE barrier function are further needed in future transplantation studies.

In this study, we used RPE-specific markers to characterize the various morphological and functional properties of these cells, including differentiation and maturation processes. Human cell markers—HNAA and STEM121 were used to localize the hRPEs in the minipig eyes in the follow-up period [41]. Bestrophin and CRALBP are typical markers of the visual cycle and late differentiation of RPEs [42,43], whose expression changed during the cultivation of hRPE cells from donors before implantation. The end-stage retinal specific neuronal markers for the rod bipolar (PKC-alpha) and the cone photoreceptors (PNA) [44] appeared to show signs of stress, yet, throughout the 8 weeks follow-up period, the retina appeared capable of maintaining its inner and outer segments intact to a great extend. This was corroborated by staining with the cell-death marker TUNEL. No positive cells could be detected in the neuroretina over the time course and only very few were detected in the implant region. The presence of reactive gliosis in the underlying retina (Iba1, Vimentin and GFAP expression) could have had beneficial effects for the retinal remodeling in our minipig study model [45,46]. It should be noted, that the results for the 8 weeks post-implantation time are from just one animal, and hence considered inconclusive for this particular time-point.

### 4.2. RPE Surgical Transplantation

Performing intraocular surgeries in vivo in primates remains challenging due to the specific features of the animal’s body and the eye in regard to general anaesthesia and surgical intervention, respectively. It requires a deep knowledge about the eye anatomy and special training on cadaver eyes of the certain species before starting with surgery on live animals. Developing new surgical approaches for treatment of retinal diseases in humans usually necessitates an experimental, large-eye animal model. Different animal species are used for experimental studies, which aim to investigate and prove the concept of subretinal injection or implantation of a variety of cell-based or gene-based therapeutic approaches. Among the most used animal models are monkeys, rabbits and pigs (or minipigs). To date, there is no standardized surgical technique for vitreoretinal and subretinal surgery in primates, as every therapeutic strategy and study protocol have been different.

In 2015, our collaborative group first reported the design fabrication of a frame-supported nanofibrous membrane for the transplantation of porcine RPEs [17]. All surgeries and implantations were performed on enucleated porcine eyes. The study results revealed the viability of the majority of the implanted RPE cells in the subretinal space. Since 2016, our group has developed and improved the surgical protocol for the subretinal implantation of a cell carrier in the eyes of minipigs in vivo. The surgical intervention applied in this study is a strictly defined and documented protocol, which was followed by every surgeon of the study group with a high success rate. Minipigs belong to a large-eye animal model, whose eye size is similar to the size of the human eye. In this context, the use of minipigs for this kind of study could be considered optimal.

In 2016, Al-Nawaiseh et al. reported a step-by-step protocol for subretinal surgery in rabbits [46]. The study described a surgical protocol based on six years’ experience with subretinal implantation of a cultured RPE monolayer on a scaffold into the subretinal space, with a detailed description of the instrumentation needed, anaesthesia, surgical technique and postoperative care. They used an implant with size of 2.4 × 1.1 mm on the uncoated 10-μm-thick polyester membrane with cultured human fetal RPE cells. The required sclerotomy was 1.4 mm in size [37,47]. In our study in minipigs as a large-eye animal model, we used a similar technique of subretinal implantation of the cell carrier. However, the characteristics of the injector, cell carrier and therefore, certain surgical steps, were different. As an example, the size of the cellular scaffold was 2.1 × 5.2 mm and the size of sclerotomy was 3.0 mm.

In 2019, Gandhi et al. reported the results of the subretinal implantation of a human fibrin hydrogel implant in pig eye in order to evaluate its degradability in vivo [48]. Previously, the authors investigated the efficacy of a fibrin hydrogel scaffold with degradation kinetics with regard to supporting the growth and differentiation of iPSC-RPE cells in vivo [49]. The study was performed on female domestic (Yorkshire) pigs cultivated on Manthei Hog Farm (Elk River, MN, USA) with a mean weight of 22–35 kg. All surgeries were performed under general anaesthesia. The size of the scaffold presented in this study was 1.5 × 5 mm. The average thickness of fibrin gels was 202.5 μm. The sclerotomy required for the insertion of the injector was 3.5 mm. The authors reported that the fibrin hydrogel membrane degraded within 8 weeks following the implantation. In our study, we used a nanofibrous carrier with an embedded supporting PET frame with a size of 2.1 × 5.2 mm. The thickness of the membrane was 3.7 µm, with a porosity of 72%. The diameter of the bead-free fibres was 380 nm, with an average pore size of 0.4 µm. Polymers such as PDLLA, with a high molecular weight can provide overall mechanical strength for a delicate nanofibrous membrane during long-term cell cultivation with degradation timeframe of at least 5 months and more [33]. The size of required sclerotomy was 3.0 mm.

In 2021, Liu et al. reported the results of a surgical transplantation of human RPE stem cell-derived RPE monolayers into non-human primates following immunosuppression [50]. In their animal model, they used 4 to 6 years old cynomolgus monkeys (Macaca fascicularis) with a weight of 3.0–5.0 kg. For immunosuppression, they used an oral administration of sirolimus, starting with the loading dose of 2 mg, and continuing with 1 mg every day at 7 days before and 3 months after the surgery. The size of the cell carrier and the required size of sclerotomy was the same described in the previous study [37,47]. In our study, we used the subcutaneous injection of tacrolimus for immunosuppression, at a low dose of 0.25 mg/kg BW. The tacrolimus blood concentrations were not measured at 6 and 8 weeks due to the 0.5 ng/mL threshold of sensitivity of our tacrolimus test kits. We suggested that the residual levels of tacrolimus in the blood (around 0.1–0.5 ng/mL) could be enough for the survival of the implanted RPE during the following 2–3 weeks because some authors described the survival of retinal xenotransplants without immunosuppression for at least 34 days (Rauer, O., Ghosh, F., 2001).

### 4.3. Injection of RPE Cell Monolayer

The successful implantation of the scaffold with cultivated cells is directly dependent on the surgical technique as well as on the instrumentation used for this purpose. To date, a small number of injector devices for the subretinal implantation of cell carriers have been described. In 2017, Fernandes et al. described the preliminary results of a new tissue injector for the subretinal implantation of an ultrathin non-absorbable substrate with cultivated cells [51]. The study results showed that the new prototype of the injector was able to efficiently deliver the tissue implant with a size of 3.5 mm to 6.0 mm in the subretinal space on the eyes of minipigs. Additionally, the results suggested that the use of the new injector allowed for a significant prevention of cell loss, reduction of the risk of tissue trauma, surgical complications and postoperative inflammation. In our study, we used a custom-designed injector, which allowed us to implant the 5.2 mm × 2.1 mm cell carrier with an adherent sheet of hRPEs. The shape of the injector possessed double bending in order to be able to place the implantation injector parallel to the retina and to reduce the risk of RPE damage during the implantation process.

## 5. Conclusions

All of the past and current protocols of choosing the right source of RPEs for transplantation studies in large-eye animal models have several disadvantages, which require improvement. The results of our study demonstrated that hRPEs could be cultivated on nanofibrous PDLLA carriers and implanted subretinally into minipigs using standard 23-gauge vitrectomy and an implantation injector. The hRPE-laden scaffolds showed relatively good incorporation into the host retina over an 8 weeks observation period, with some indication of a gliotic scar formation, and a likely neuroinflammatory response in the transplanted area beside the use of systemic immunosuppression. Further studies are needed for the exact verification of RPE-implant conditions at eight-week timepoint upon RPE implantation, with an additional modification of the immunosuppression protocol.

## Figures and Tables

**Table 1:** Experimental setup and timeline.

Pig Number	Time of Euthanasia (Weeks)	Right Eye	Left Eye	Time of OCT Scans and Fundus Photography (Weeks)
4 (T149)	1	Treated eye	Control eye	1
1 (T141)	2	Treated eye *	Treated eye	1, 2
3 (T146)	6	Treated eye	Control eye	1, 2, 6
6 (T192)	6	Treated eye	Control eye	1, 2, 6
5 (T145A)	8	Treated eye	Control eye	1, 2, 6, 8

* The treated eye had a subretinal bleeding during the procedure.

**Table 2:** Antibodies used for the immunohistochemical analysis.

Protein	Source	Manufacturer	Working Dilution
HNAA	mAb mouse	*Novus Biologicals*, UK, Abingdon, (235-1) NBP2-34342-0.1 mg	1:300
STEM121	mAb mouse	*Takara Bio Inc.*, Kusatsu, Japan, cat. No. Y40410	1:100
Bestrophin	mAb mouse	*Santa Cruz Inc.*, Germany, Heidelberg, cat. No. sc-32792	1:50
CRALBP	mAb mouse	*Novus Biologicals* UK, Abingdon, (B2) cat. No. NB100-74392	1:100
Vimentin	mAb mouse	*Cell Signaling*, (D21H3), USA, cat. No. 5741	1:100
GFAP	pAb rabbit	*Merck Millipore*, Germany, Darmstadt, rabbit polyclonal antibody, cat. No. AB5804, lot No. 2464502	1:1000
IbaI, Microglial Marker	mAb mouse	*Abcam*, Cambridge, UK, mouse monoclonal antibody, cat. No. ab15690, clone 1022-5	1:250
PKC alpha	pAb rabbit	*Sigma-Aldrich*, Germany, Taufkirchen, rabbit polyclonal, cat. No. P4334	1:10,000
Lectin PNA Conjugate Alexa Fluor 488		*Molecular probes*, cat. No. L-21409	1:300

## Data Availability

All data related to this work can be made available upon request to the corresponding authors.

## Supplementary Materials

The following supporting information can be downloaded at: https://www.mdpi.com/article/10.3390/biomedicines10030669/s1, Figure S1: Position of the 23-gauge (G) trocars, which were inserted 2.5–3 mm from the limbus; Figure S2: TUNEL assay in the implant area and underlying neuroretina (D, H, L, P, T) followed up to 8 weeks.
