# Supplementary material for: Subretinal Implantation of Human Primary RPE Cells Cultured on Nanofibrous Membranes in Minipigs
Source: Biomedicines. 2022 Mar 14;10(3):669. doi: 10.3390/biomedicines10030669 (PMC8945676; doi:10.3390/biomedicines10030669)
Supplement: Supplementary file 1 [file biomedicines-10-00669-s001.zip › biomedicines-1597320-supplementary.pdf]

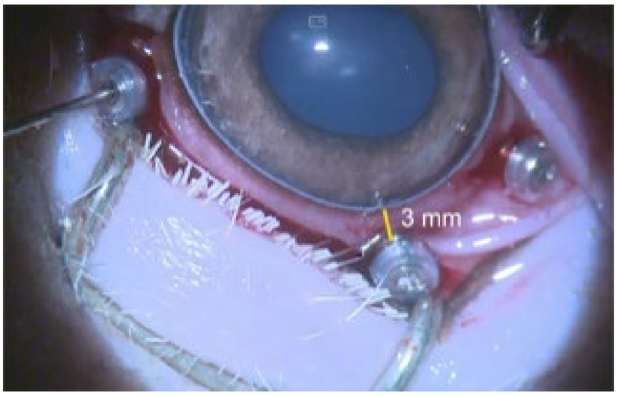

Supplementary Figure S1. Position of the 23-gauge (G) trocars, which were inserted 2.5-3 mm from the limbus.

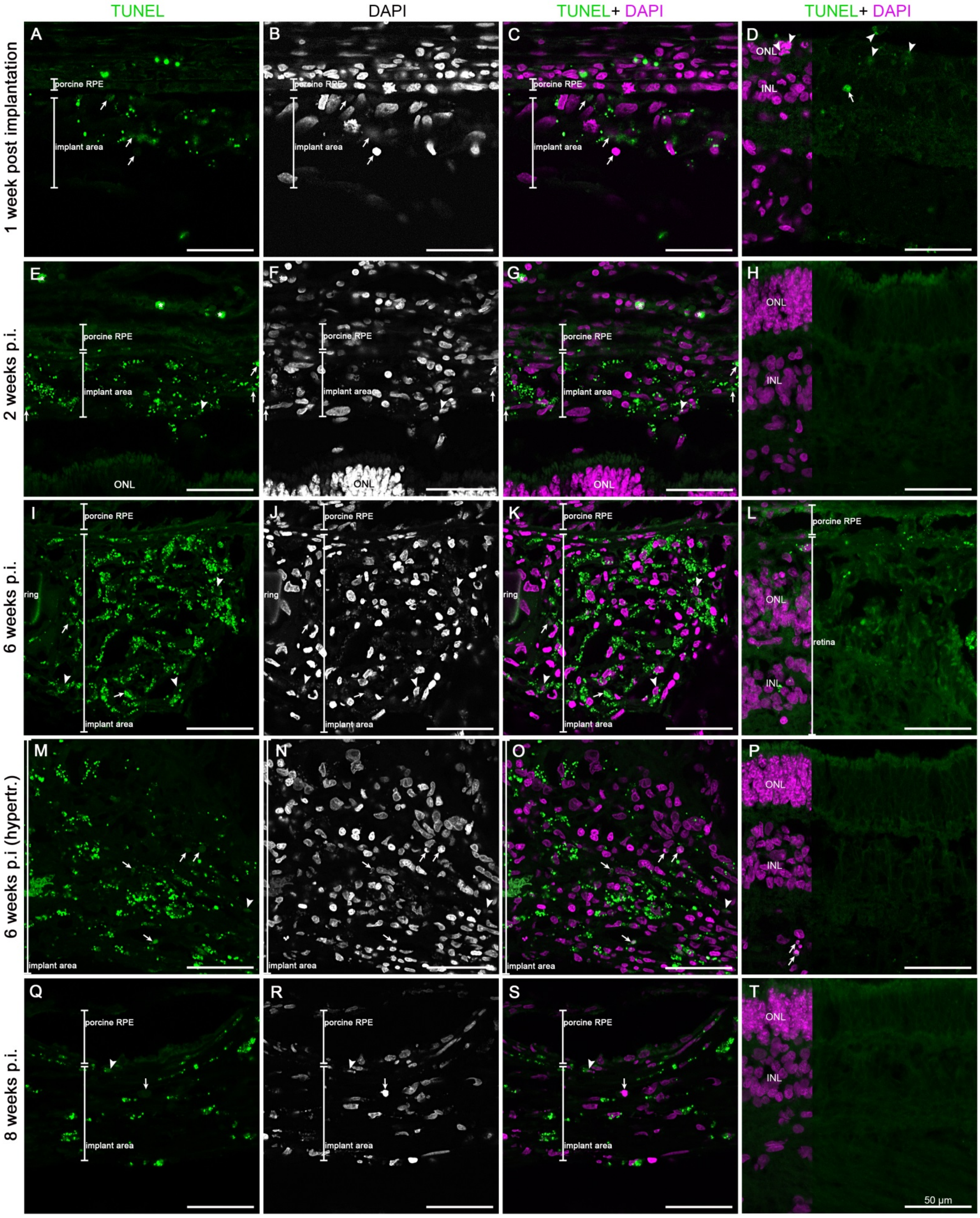

Supplementary Figure S2. TUNEL assay in the implant area and underlying neuroretina (D, H, L, P, T) followed up to 8 weeks.
